# Imaging Calcium in Hippocampal Presynaptic Terminals With a Ratiometric Calcium Sensor in a Novel Transgenic Mouse

**DOI:** 10.3389/fncel.2018.00209

**Published:** 2018-07-19

**Authors:** Ibrahim Al-Osta, Mariusz Mucha, Daniel Pereda, Marta Piqué-Gili, Albert E. Okorocha, Roisin Thomas, Nicholas A. Hartell

**Affiliations:** Department of Neuroscience, Psychology and Behaviour, University of Leicester, Leicester, United Kingdom

**Keywords:** presynaptic terminals, calcium imaging, transgenic mice, hippocampus, CA1, CA3, long-term potentiation

## Abstract

Genetically encoded calcium indicators (GECIs) have gained widespread use for measurement of neuronal activity but their low expression levels in transgenic mice tend to limit sensitivity. We have developed a transgenic mouse line (SyG37) that expresses a ratiometric calcium sensor, SyGCaMP2-mCherry, that is expressed throughout the brain but targeted to presynaptic terminals. Within the CA1 and CA3 regions of hippocampus of male and female mice, SyGaMP2 fluorescence responds linearly up to 10 electrical stimuli at frequencies up to 100 Hz and it can detect responses to a single stimulus. Responses in single boutons can be measured using multiphoton microscopy. The ensemble amplitude of SyGCaMP2 responses is a function of the number of stimuli applied and the number of contributing boutons. The peak responses and initial rates of calcium influx in single boutons in CA1 and CA3 were similar but the rate of calcium clearance from CA3 boutons after stimulation was significantly faster. In CA1, DNQX reduced SyGCaMP2 responses to Schaffer collateral stimulation to 86% of baseline indicating that 14% of the total response originated from presynaptic terminals of neurones synaptically driven via AMPA receptors. Theta burst stimulation induced long-term potentiation (LTP) of both SyGCaMP2 and fEPSP responses in both young and 18-month-old mice. The proportion of postsynaptically connected terminals increased significantly to 76% of the total after LTP induction. The SyG37 mouse allows stable optical detection of synaptic activation and connectivity at the single bouton level and can be used to characterize the contributions of presynaptic calcium to synaptic transmission and plasticity.

## Introduction

Genetically encoded calcium indicators (GECIs) have gained widespread use for measuring neuronal activity. They can be targeted to individual neuronal regions, cell populations and even to specific intracellular compartments (Tian et al., 2012). Viral delivery provides a relatively convenient method for population specific delivery but expression levels can be variable, decline with time, and not all neurones or cell populations are equally amenable to particular viral delivery and packaging methods. The development of transgenic mice that express calcium indicators is therefore of interest. Although transgenic mice can produce stable and reproducible expression over long periods of time, their expression levels are often much lower than those achieved with viral transduction leading to lower sensitivity. The development of brighter, more sensitive calcium indicators, such as the GCaMP family (Nakai et al., 2001; Tallini et al., 2006, 2007; Tian et al., 2009; Akerboom et al., 2012; Chen et al., 2013) has helped to overcome these limitations and mice expressing various members of this family of GECIs have been used to detect neuronal activity in vertebrate and invertebrate systems *in vivo* (Tallini et al., 2006; Tian et al., 2009; Zariwala et al., 2012; Dana et al., 2014). Sensitivity can be further improved by targeting calcium indicators to subcellular microdomains where calcium levels within compartments of restricted volume which undergo rapid and substantial changes in calcium concentration. For example, whereas GCaMP2 lacks the sensitivity to detect single action potentials when expressed non-specifically in drosophila neurones (Hendel et al., 2008), it has been used to detect responses in cerebellar parallel fibers through cell specific expression in granule cells (Díez-García et al., 2005) whose presynaptic terminals have very low volumes. Whereas this approach allowed detection in synchronized bundles of activated fibers, specific targeting of GCaMP2 to presynaptic terminals by fusing it to the extracellular facing terminus of the vesicular protein synaptophysin allowed detection of single action potentials (Dreosti et al., 2009) in single, identified presynaptic terminals. Whilst GCaMP2 is not as bright or sensitive as the more recently developed GCaMP6 family of GECIs, it has a much faster response time to calcium (Chen et al., 2013).

A calcium indicator expressed at presynaptic terminals has the potential to allow detection of neuronal activity at the level of single synapses and to allow optical observation and quantitative measurements of the presynaptic contributions to synaptic transmission even when transmission itself is blocked. In combination with electrophysiological recordings it can be used to help establish the origin and mechanisms of changes in synaptic transmission strength. We have created a transgenic mouse using a ratiometric GECI created by combining SyGCaMP2 with the red fluorescent protein mCherry, expressed under the control of the non-specific, neuronal Thy1–2 promotor. The addition of a red fluorescent protein was designed to facilitate detection of synaptic boutons and to permit ratiometric measurement of calcium. Here we characterize the expression patterns and response characteristics of a mouse strain referred to as SyG37 and use it to compare patterns of calcium signaling in presynaptic terminals in the CA1 and CA3 regions of the hippocampus. This mouse model can be used to record long-term potentiation (LTP) optically in the CA1 region of the hippocampus, in both young and aged mice. The SyG37 transgenic mouse therefore allows non-invasive, stable, optical detection of synaptic activation and connectivity at the single bouton level and can be used to characterize the patterns and extent of fiber activity and connectivity in response to electrical activation and the contributions of presynaptic calcium to synaptic transmission and plasticity.

## Materials and Methods

### Generation of SyGCaMP2-mCherry Mice

SyGCaMP2-mCherry was created by fusing mCherry to the C-terminus of SyGCaMP2 which is in turn a fusion of GCaMP2 to the C-terminus of Synaptophysin-1 (Dreosti et al., 2009). The red fluorescent protein served two roles. First, it facilitated visualization of presynaptic terminals since GCaMP2 is only weakly fluorescent at resting calcium concentrations (Tallini et al., 2006). Second, it could be used as a reference to allow quantification of expression levels and hence measurement of absolute calcium concentrations. Transgenic mice were created on a C57 Blk6 background using the Thy1–2 promotor which produces founder-dependent neuronal expression that appears from P7 to P10 onward and which is stable throughout adulthood (Caroni, 1997). Primers for genotyping were targeted to the fluorescent proteins within SyGCaMP2 (forward primer 5′-CGACAACCACTACCTGAGCA; reverse primer 5′-GAACTTCAGGGTCAGCTTGC). The cytidine monophospho-N-acetyl-neuraminic acid hydroxylase (Cmah) forward primer (5′-CAGCTTGCTTATCACGTGTG) and Cmah reverse primer (5′-TGGTGCTCACGTCTAACTTC) were used as controls to ensure that DNA was recovered from the samples.

### Preparation of Solutions and Brain Slices

Mice of both sexes, aged between 2 months and 18 months, were culled in accordance with Home Office regulations and with local ethical approval and the brains quickly removed and placed in an ice cold artificial cerebrospinal fluid (aCSF) consisting of (in mM); 127 NaCl; 1.25 KH_2_PO_4_; 1.30 MgSO_4_.7H_2_O; 26 NaHCO_3_; 1.61 KCl; 10 glucose, equilibrated with 95% O_2_–5% CO_2_ to pH 7.4. Transverse hippocampal slices, 300 μm thick, were prepared using a vibratome (Dosaka EM, Kyoto, Japan) and equilibrated at room temperature in the same solution for at least 1 h prior to experiments.

### Epifluorescence Imaging and Electrophysiological Data Acquisition

Slices were placed in a custom-built perfusion chamber on the stage of an upright microscope (Olympus, Tokyo, Japan). A flattened silver ring with nylon fibers attached at 1–2 mm intervals was used to hold the slices in place. Glass stimulating electrodes with tip resistances of ~2 MΩ when filled with aCSF were fabricated using a micropipette puller (P-97, Sutter). Electrodes were connected to an isolated stimulator (Digitimer Ltd., Welwyn Garden City, UK) and their tips placed onto the surface of the slice in the *stratum (s.) radiatum* between CA3 and CA1 to activate Schaffer collateral-associational commissural (SC-AC) fibers. A second aCSF-filled micropipette, with a resistance of between 2 MΩ and 4 MΩ, was placed midway in the *s. radiatum* within CA1 to measure extracellular excitatory field potentials (fEPSPs) with a patch clamp amplifier (Axopatch 200B, Molecular Devices, Sunnyvale, CA, USA). Transmission and fluorescence images were obtained using a Flash EM or QuantEM EMCCD camera (Hamamatsu, Hamamatus, Japan; Photometrics, Tucson, AZ, USA). For experiments carried out in the CA3 region of the hippocampus, stimulating electrodes were placed in the *s. granulosum* of the *dentate gyrus* and the recording electrode was placed in the *s. lucidum* of CA3. Fluorescent light was supplied with a custom-made LED light source or a monochromator (Till Photonics GmbH, Graefelfing, Germany). A dual excitation, double dichroic filter set designed for EGFP and mCherry (59022; Chroma Technology, Bellows Falls, VT, USA) was used excite SyGCaMP2-mCherry and collect the green and red signals from each of its fluorophores. The calcium sensor GCaMP2 was activated at 470 ± 20 nm and its emission collected at 520 ± 20 nm. mCherry was activated at 570 ± 20 nm and its emission collected at 630 ± 30 nm.

Images were collected using software routines written using Igor Pro 6 (Wavemetrics Inc. Lake Oswego, OR, USA). The camera was controlled using SIDX 6 (Bruxton Corporation, Seattle, WA, USA) which is a camera driver for Igor Pro. Image acquisition was synchronized with light excitation, electrical stimulation and recordings using an M-Series DAQ board (National Instruments, Austin, TX, USA), also controlled with Igor Pro using Nidaqmx tools for Igor. Stacks of images were collected whilst the light source was alternated between blue and yellow excitation wavelengths. This produced a stack of interleaved images which were separated into separate stacks offline. WinLTP software (Anderson and Collingridge, 2007) was used in combination with a Digidata 1322A digitizer (Molecular Devices) or M-Series DAQ board (National Instruments) for concurrent electrophysiological recordings and to control electrical stimulation patterns and to trigger the imaging. The effects of stimulus intensity on SyGCaMP2-mCherry and field potential responses were routinely measured. Imaging responses under identical conditions but at an intensity of zero volts were collected in each experiment to isolate the kinetics of sensor photobleaching so that this could be compensated for in subsequent recordings. A stimulus intensity of 20 volts was generally used for experiments as this intensity produced a clear but sub-maximal response in all slices. At this fixed but sub-maximal intensity, the effects of stimulation frequency between 1 Hz and 100 Hz were measured; the effects of changing the stimulus number over a range between 1 and 100, at a fixed frequency of 20 Hz, were then assessed.

For LTP experiments, pairs of stimuli were delivered every 10 s at an interval of 50 ms to assess the level of paired pulse facilitation of fEPSPs. Once a stable baseline response had been established, theta burst stimulation was applied which consisted of 20 trains of four stimuli delivered at 100 Hz, repeated at an interval of 350 ms. The extent and incidence of LTP within each age group of animals was then assessed. These experiments were performed at 36°C. Imaging data were collected as described above at 5-min intervals during periods of baseline, theta burst stimulation and after theta burst stimulation.

### Image Analysis

Stacks of interleaved SyGCaMP2 and mCherry images were separated and regions of interest (ROIs) were placed over defined areas of the slices and changes in absolute fluorescence measured over time. Photobleaching effects were compensated for by measuring fluorescence in a set of images collected without synaptic activation. The decline in fluorescence was fitted with a double exponential function. The time constants for photobleaching were measured and used to remove the effects of bleaching on stimulated responses. The mean fluorescence values for each bleach compensated ROI prior to electrical stimulation were calculated (F_0_) and the absolute fluorescence expressed as a proportion of this (F/F_0_). The last measurement before stimulation was taken as time zero and data were plotted relative to the onset of stimulation. Measurements from ROIs within areas such as *s. oriens*, *s. pyramidale* and *s. radiatum* were averaged together. Measurements of the peak amplitude of the responses, time to peak, the initial slope, time constant of decay and area under the curve were made from each image stack and then these values pooled from several experiments and the means and standard errors of the mean (SEM) plotted against time. Since SyGCaMP2 fluorescence decay after stimulation could follow either a single or double exponential process in no obvious age-related pattern, the fast phase of decay was measured as either a single exponential or the faster of two time constants where a double exponential decay was observed.

In widefield epifluorescence measurements at low magnification, we could not separate SyGCaMP2-mCherry fluorescence originating from densely packed boutons from background autofluorescence and so we did not routinely background subtract fluorescence signals before performing our F/F_0_ calculations. This introduces a systematic underestimation of the true dynamic signal but since SyGCaMP2-mCherry expression was extremely consistent between animals of a similar age and autofluorescence was comparatively low, we did not routinely correct for this. However, where comparisons were made between responses in different hippocampal regions we estimated background fluorescence within each region from aged matched wild-type (WT) animals to ensure that region specific differences in the absolute size of responses were not due to background subtraction errors.

### Single Bouton Imaging

Images of single boutons were obtained at 920 nm using a Zeiss MP7 multiphoton microscope (Carl Zeiss Microscopy GmbH, Jena, Germany) equipped with a 20× 1.0 NA objective and an optical zoom of 3. Stacks of images comprising 512 pixels by 64 lines were collected at intervals of 98 or 110 ms. An Arduino Uno was used to trigger image collection and electrical stimulation as described above. Trains of 10–20 stimuli were delivered at a frequency of 20 Hz and the effects of stimulus intensity from 0 V to 70 V tested.

Single bouton responses were analyzed using Igor Pro with custom routines and those from a package of semi-automated routines designed for measuring calcium responses from presynaptic boutons (for a detailed description, see Dorostkar et al., 2010). ROIs were identified by thresholding background subtracted images constructed by averaging over time each stack of 100 images per stimulus condition. Averaged images were transformed using the Laplace operator and then a multiple of the standard deviation (SD) of all the pixel values in the Laplace operator used as a threshold. Changes in fluorescence over time from each identified ROI were then converted into F/F_0_ values and each plotted as single lines to create a 2D image illustrating stimulus dependent changes in fluorescence over time. A pseudo-color look-up table was applied to highlight effects. A semi-automated hierarchical clustering analysis was then used to categorize each ROI as either responding to stimulation or not responding. The results of this clustering were verified manually and adjustments made if required. The number of ROIs per stimulus condition that responded to stimulation was then recorded as a proportion of the total number of regions identified. F/F_0_ responses for responding regions were pooled together to create an average response for responders under each stimulus condition. To illustrate the positions of responding and non-responding puncta, green and red dots were plotted on the original images used for thresholding centered at the point of maximum intensity for each ROI. The effectiveness of this approach was validated against a manual approach that yielded almost identical results except that far fewer ROIs per field of view were identified by eye. Averages of images taken before stimulation were used to create F/F_0_ images using averages of images taken during stimulation at the maximum intensity tested of 40 V. This revealed the position of boutons that responded at this maximum intensity. These locations were then used to define 3 × 3 pixel ROIs that just circumscribed these single, responding boutons. These ROIs were used to measure responses at the other intensities tested along with a separate group of neighboring ROIs that were used to measure the background fluorescence. Fluorescence responses were background subtracted and converted into F/F_0_ values and the peak responses, slopes and areas under the curve for 2 s after stimulation calculated. Individual bouton responses at each intensity were manually categorized as responding to stimulation or not and the proportions at each stimulus intensity or number measured. The response of the entire frame was also background subtracted and F/F_0_ values measured over time to show the average response.

### Image Stitching

Images of whole brain sections were taken using an epifluorescence microscope equipped with an sCMOS camera (Prime; Photometrics) with a ×4 air immersion objective (NA 0.2). Slices were placed on a section of insert designed for use with organotypic cultures and perfused slowly with oxygenated aCSF during the imaging process. This thin, microporous layer minimized slice movement during image collection and aided oxygenation. Overlapping Images of red and green fluorescence were taken over the entire section. Prior to stitching, an average of image of all sections without edges was taken and filtered with a Gaussian filter with a kernel of 121 pixels to remove any high frequency information. This average and filtered image was then divided by the average fluorescence to produce a reference image that provided a good approximation of the illumination pattern of the excitation source *in situ*. Individual images were then divided by the filtered reference image. This approach “flattened” the uneven light intensity of the individual images while maintaining the quantitative nature of the data. Green and red sets of images were then stitched together using the stitching plugin (Preibisch et al., 2009) within FIJI (Schindelin et al., 2012) and displayed with identical brightness settings using Igor Pro. The ratio of green to red images was calculated by first background subtracting mean fluorescence values measured from WT brain slices and then dividing green images by red images. The ratio images were displayed using a look up table with identical brightness settings. The absolute ratio depends upon the relative intensities of light used to excite SyGCaMP2 and mCherry. This was kept consistent within recording setups but varied between microscopes.

### Immunohistochemistry

For immunohistochemistry experiments, mice were deeply anesthetized with intraperitoneal sodium pentobarbital and transcardially perfused with normal saline containing protease inhibitors (cOmplete™ protease inhibitors, Roche, Basel, Switzerland), followed by 4% paraformaldehyde in phosphate buffer. After perfusion, the brains were removed and post-fixed overnight at 4°C and then rinsed three times in tris-HCl and incubated for 12 h in 20% sucrose in tris-HCl of pH 7.2. The brains were removed and cut into two hemispheres. Each hemisphere was embedded in Surgipath FSC22 clear freezing medium (Leica Biosystems, Milton Keynes, UK) in the desired orientation and fast frozen in liquid nitrogen. Transverse hippocampal slices of 10–20 μm thickness were obtained from SyG37 and WT mice using a cryostat (OTF-5040; Bright Instruments, Luton, UK). To quench autofluorescence, slices were incubated in 0.1 M glycine in PBS for 1 h and washed with 0.1 M NH_4_Cl in PBS for 1 min. Slices were then incubated for 1 h at room temperature in tris-buffered saline (TBS) with 0.1% Triton X-100 (TBS-T) and 10% normal goat serum (NGS) or donkey serum. Slices were then incubated in primary antibodies in TBS-T and 10% NGS overnight at 4°C. Slices were rinsed in TBS, incubated with secondary antibodies, then rinsed in TBS and mounted on super-frost glass slides (VWR International, Radnor, PA, USA). Sections in which primary antibodies were omitted were used as controls. The primary antibodies used were anti-vGLUT1 guinea pig polyclonal (1:1000; Synaptic Systems GmbH, Goettingen, Germany Cat# 135 304), anti-mCherry goat polyclonal (1:1000; SICGEN, Cantanhede, Portugal, Cat# AB0040-200), anti-bassoon rabbit polyclonal (1:1000; Abcam, Cambridge, UK, Cat# 141 002), anti-PSD-95 rabbit polyclonal (1:1000; Abcam Cat# ab18258), and anti-vGAT mouse polyclonal (1:1000; Synaptic Systems Cat# 131 011BT). The secondary antibodies used were goat anti-guinea pig Alexa Fluor 488 (1:2000; Thermo Fisher Scientific, Cramlington, UK, Cat# A-11073), donkey anti-goat Alexa Fluor-594 (1:2000; Thermo Fisher Scientific Cat# A-11058), donkey anti-rabbit Alexa Fluor-488 (1:2000; Thermo Fisher Scientific Cat# A-21206), and goat anti-mouse Alexa Fluor-546 (1:2000; Thermo Fisher Scientific Cat# A-11001). Sections were examined with a multiphoton microscope (LSM 7 MP; Carl Zeiss) equipped with green and red fluorescence filter sets with a water immersion objective (×20, 1.0 NA) using ZEN software (ZEN Digital Imaging for Light Microscopy, Carl Zeiss). Series of images from hippocampal slices were taken in the axial plane (z-stacks) at a pixel resolution of 1024 × 1024 and a stack of at least five images was ultimately created.

### Experimental Design and Statistical Analysis

Experiments were carried out on brain slices from both male and female SyG37 and wild type (C57 Blk6) mice. Data are presented as mean values along with the SEM. The numbers of replicates for experiments for each experiment are recorded in the text or in figure legends along with the levels of statistical significance. No more than two replicates were obtained from any one animal and replicates represent data from separate brain slices. The non-parametric Mann-Whitney U-test (for unpaired data) or Wilcoxon test (for paired data) were used to test for statistical significance between two data sets. The Friedman’s (for unpaired data) or Kruskall-Wallace (for paired data) test with Dunn’s multiple comparison test were used to test for statistical differences between multiple groups of data. For co-localization analysis, a Pearson correlation coefficient was calculated for the co-variation of fluorescence from pairs of antibodies along lines drawn through immunohistochemically stained sections.

## Results

### Expression of SyGCaMP2-mCherry in SyG37 Mice

**Figure 1:**
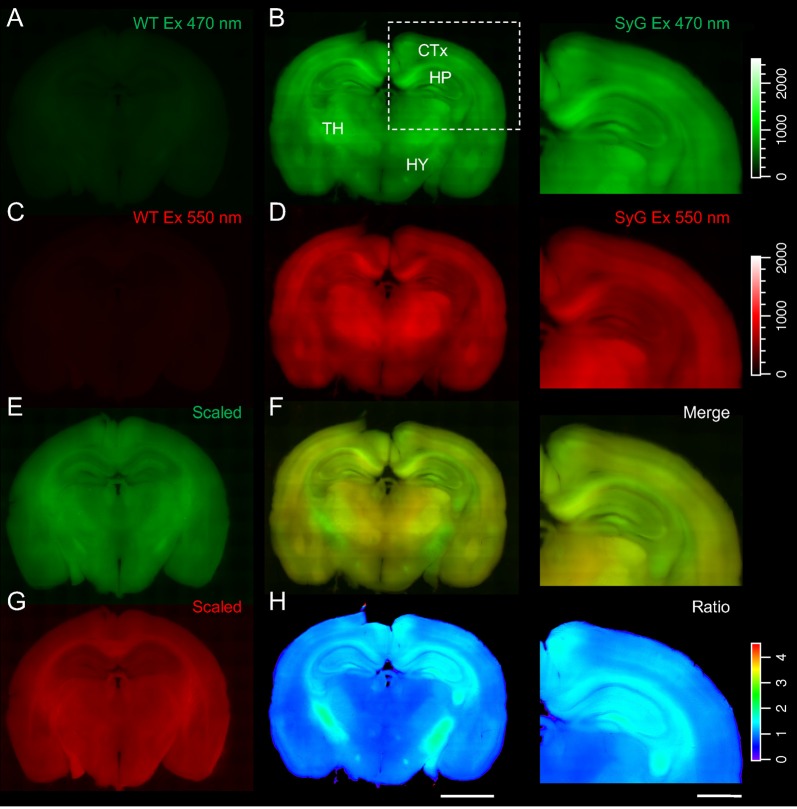
Global expression patterns of SyGCaMP2-mCherry in SyG37 mouse brain. Arrays of epifluorescence images were taken from living, 400 μm thick coronal slices prepared from adult, wild-type (WT; left) and SyG37 mice (center and right) and stitched together as described in the methods section. The first two rows show images collected with excitation wavelengths of 470 **(A,B)** and 550 nm **(C,D)** using identical brightness settings for red and green images for quantitative comparison. Panels **(E,G)** show images of the same WT slices illustrated in panels **(A,C)** but with the brightness increased to reveal autofluorescence detail. Panels **(F,H)** shows the images in **(B,D)** merged and the SyGCaMP2 to mCherry fluorescence ratio respectively. A cold-hot look up table was applied to highlight differences in ratio. The right-hand panel of images show the cortical and hippocampal structures outlined with the dotted box in panel **(A)** magnified. The scale bars in panel **(H)** represent 2 mm (left) and 1 mm (right) respectively. Calibration bars for all images, with the exception of those in panels **(E–G)** which were scaled differently for illustrative purposes, are shown to the right-hand side.

The expression patterns of the SyGCaMP2-mCherry sensor in living 400 μm thick coronal slices of SyG37 mice were compared with slices obtained from aged matched, WT mice. Arrays of images of SyGCaMP2 and mCherry fluorescence were taken using a low magnification (×4) objective lens and stitched together to make a montage of the whole brain. The results are summarized in Figure 1. Both green and red fluorescence were markedly brighter in SyG37 mice compared to WT animals indicating that both the green and red fluorescent protein components of SyGCaMP2-mCherry were expressed. mCherry, whose fluorescence is calcium-independent, and therefore a direct indicator of expression levels, was present throughout the brain but was noticeably high in the subiculum of the hippocampus, deeper layers of the cortex, the hippocampus, thalamus and fiber tracts, especially those surrounding the hippocampus including the fornix, corpus callosum and fimbria.

### Characteristics of CA1 Responses to CA3 Stimulation

**Figure 2:**
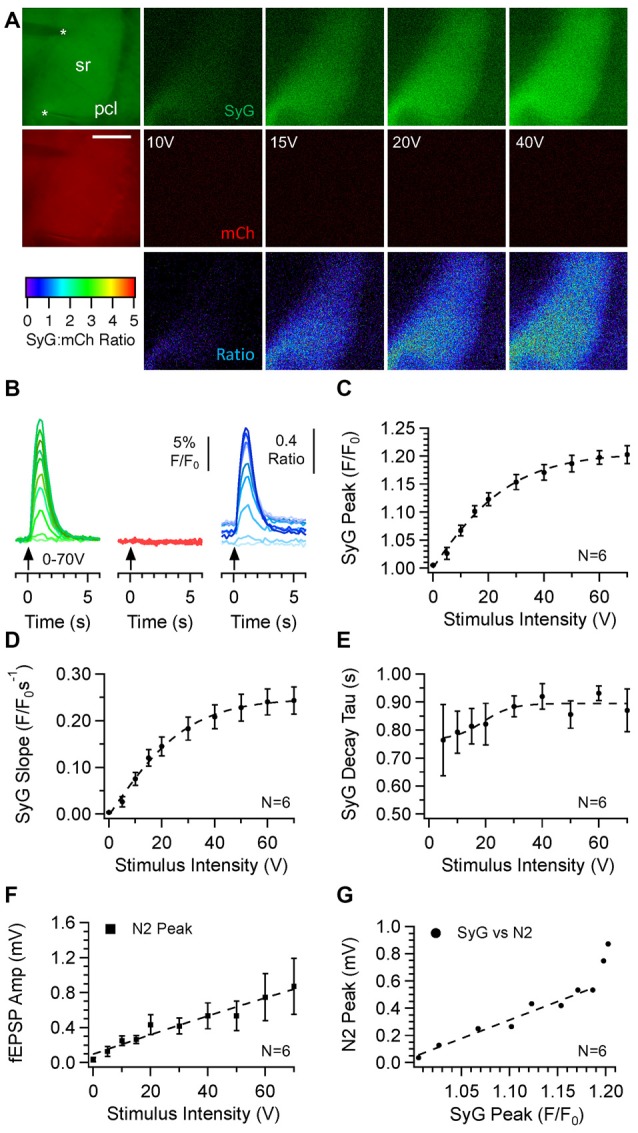
The effects of stimulus intensity on optical and electrophysiogical responses recorded from the CA1 region of the hippocampus in SyG37 mice. Bursts of 20 stimuli over a range of intensities were delivered at 20 Hz via a patch pipette placed in the *s. radiatum* of CA3. Images of SyGCaMP2 (green) and mCherry fluorescence (red) are shown along with the ratio of the two and images illustrating the difference in fluorescence for each fluorophore and their ratio before and during stimulation at each intensity labeled **(A)**. The positions of the recording and stimulating electrodes are marked with upper and lower asterisks respectively. Abbreviations: pcl; pyramidal cell layer; sr; *s. radiatum*. The horizontal scale bar represents 100 μm. Responses over time are shown **(B)** for SyGCaMP2 and mCherry fluorescence extracted from regions of interest (ROIs) placed over the *s radiatum (sr)*. The ratios of SyGCaMP2:mCherry fluorescence at each intensity are shown in blue. Increasing intensities are depicted with darker hues. The mean and standard errors of the mean (SEM) peak **(C)**, initial slope **(D)** and decay time constant **(E)** of SyGCaMP2 fluorescence responses and the N2 component of fEPSPs **(F)** are plotted against intensity. The relationship between SyGCaMP2 and N2 peak responses is shown in panel **(G)**. A line of best fit was plotted for values measured below 50 V where the relationship was linear. Data were obtained from six separate hippocampal slices taken from four different mice.

We first characterized SyGCaMP2 responses within the CA1 region of the hippocampus to electrical stimulation of CA3 (Figure 2). Stimulating electrodes were placed in the *s. radiatum* of CA3 and fluorescence measured in CA1. Bursts of 20 stimuli, delivered at 20 Hz, were applied over a range of stimulus intensities and measurements made from ROIs placed over CA1 *s. radiatum*, *s. pyramidale* and *s. oriens*. A typical pattern of activation is shown in Figure 2A. Responses were characteristically largest in the *s. radiatum* and smaller in the *s. pyramidale* and *s. oriens*. In most cases, a clear band of activity within the *s. radiatum* was evident. With increasing intensity, responses increased in amplitude. Simultaneous measurements of mCherry fluorescence showed no stimulus related changes. Measurement of the ratio of SyGCaMP2 to mCherry showed the same effect but also revealed small changes in absolute baseline ratio during the experiment (Figures 2A,B).

Although the stimulus response characteristics might be affected by numerous parameters including slice health, animal age and stimulating electrode placement and characteristics, the basic patterns and magnitudes of peak amplitudes and initial slopes of responses to increasing stimulus intensity were very consistent between experiments (Figures 2C,D) and followed a sigmoidal relationship. Responses recovered rapidly to baseline levels immediately after stimulation. The single exponential decay time constants increased slightly with stimulus intensity but reached a plateau after 30 V (Figure 2E). Simultaneously recorded field potentials also increased with stimulus intensity (Figure 2F). The N2 component of the field potential and the peak SyGCaMP2 response showed a strong linear correlation up to 50 V but at higher intensities the fluorescence signal started to saturate (Figure 2G).

**Figure 3:**
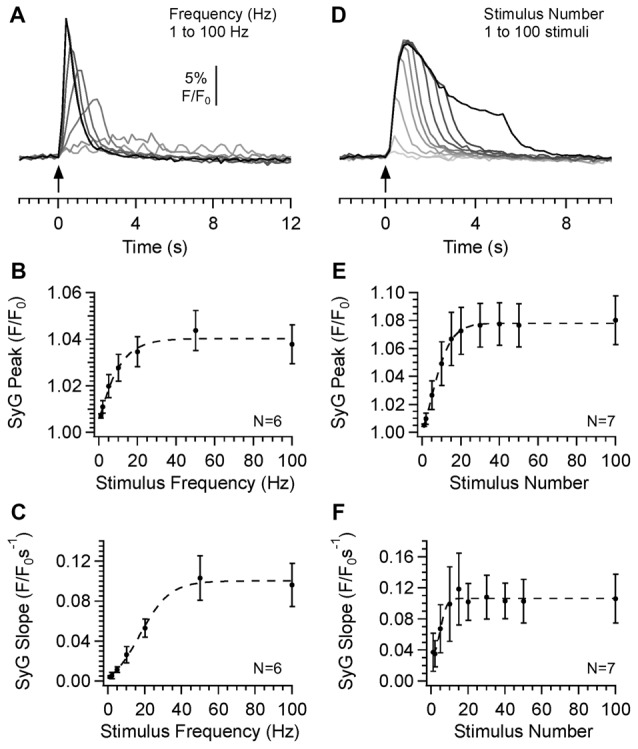
Effect of stimulus frequency and number on responses recorded from the *s. radiatum* of the CA1 region of SyG37 mice. Trains of 10 stimuli were applied at a fixed voltage at frequencies between 1 Hz and 100 Hz. In panel **(A)** responses over time are shown for SyGCaMP2 fluorescence extracted from ROIs placed over the *s radiatum (sr)*. Darker gray levels are used to distinguish higher frequencies of stimulation. The means and SEM for the peak **(B)** and initial slopes **(C)** of responses are shown (*n* = 6 slices from four animals). Panels **(D–F)** illustrate the effects of changing the number of stimuli, delivered at a fixed intensity and frequency of 20 Hz. Darker gray levels are used to distinguish the effects of more stimuli (*n* = 7 slices from four animals).

Increasing the stimulus frequency at a fixed stimulus number and intensity produced an increase in peak response size which was roughly linear up to 10 Hz and then started to plateau with a maximum at 50 Hz (Figures 3A,B). At frequencies of 1 and 2 Hz, it was possible to identify responses to individual stimuli (Figure 3A). Measurement of the initial slope showed a similar trend (Figure 3C) with the maximum occurring at 50 Hz. Figures 3D–F show the effects of increasing the number of stimuli delivered at 20 V with a fixed frequency of 20 Hz. The peak and slope responses were similar; responses increased approximately linearly up to 20 stimuli after which they started to plateau.

### Immunocytochemical Localization of SyGCaMP2-mCherry to Presynaptic Terminals

These results demonstrate that in SyG37 mice, within the CA1 region of the hippocampus, it is possible to measure robust responses to electrical activation of SC-AC fibers. By linking GCaMP2 to synaptophysin, we expected expression to be restricted to presynaptic terminals as previously shown (Dreosti et al., 2009). Using widefield epifluorescence imaging, we found no evidence for expression in cell bodies or other post-synaptic structures but the high density of expression prevented identification of individual synaptic boutons. We therefore examined the expression patterns of SyGCaMP2-mCherry expression in SyG37 mice using multiphoton microscopy and immunocytochemical co-localization of an antibody raised against mCherry with antibodies against known pre- and post-synaptic markers. In these studies, we did not use the natural fluorescence characteristics of SyGCaMP2-mCherry because the fixation process reduced fluorescence significantly. We were able to minimize the interference of any remaining natural fluorescence because the two photon excitation wavelengths for the Alexa-labeled secondary antibodies (780 nm) is distant from GCaMP2 which was activated at 920 nm. mCherry can be activated at wavelengths below 800 nm (Drobizhev et al., 2011) but we used a red secondary antibody so that the natural fluorescence of mCherry would coincide with activation of the secondary antibody. Antibody selectivity and the absence of signal cross-talk was confirmed with a series of control experiments using WT mice and by systematically omitting primary and secondary antibodies to check for primary and secondary antibody specificity.

**Figure 4:**
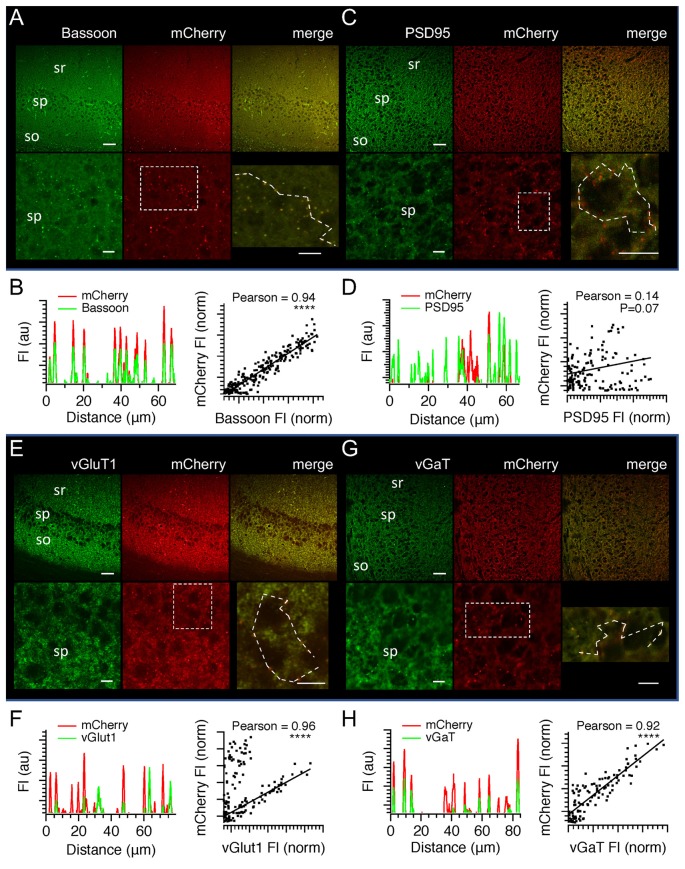
Multiphoton images illustrating the immunocytochemical location of SyGCaMP2-mCherry in the CA1 region of the hippocampus. An antibody raised against mCherry was used to establish the location of SyGCaMP2-mCherry with respect to the presynaptic marker bassoon **(A,B)** and the postsynaptic marker PSD-95 **(C,D)**. Images in green show the expression patterns of bassoon and PSD-95 and images in red illustrate the location of mCherry. The third column shows a merged image of green and red fluorescence in each case. Scale bars represent 50 μm in the upper and 10 μm in the lower rows. The positions of the *s. radiatum* (sr), *s. pyramidale* (sp) and *s. oriens* (so) are indicated. Panels **(B,D)** illustrate fluorescence values extracted from lines drawn through punctate regions of the higher magnification images illustrated in panels **(A,B)** respectively. Absolute fluorescence values are not shown since they were dependent on both the microscope settings for each fluorophore and the relative proportion of the merge value selected. To the right are graphs showing the relationship between each synaptic marker and mCherry along with the Pearson correlation and *P* values in each case (****P < 0.0001). Panels **(E–H)** illustrate images obtained using antibodies against vGluT1 and vGaT which identify glutamatergic and GABA-ergic terminals respectively. Data are presented in the same way as panels **(A–D)**.

Expression patterns of bassoon were indistinguishable from those of mCherry in SyG37 positive mice (Figure 4A). mCherry fluorescence was absent in WT mice but bassoon fluorescence expression patterns were identical to those in SyG37 positive mice (data not shown). Within the *s. radiatum*, fluorescence was dense but clearly punctate at higher magnifications. In the *s. pyramidale*, there was no evidence for expression of either mCherry or bassoon within cell bodies but puncta were evident around soma. A correlation of green and red fluorescence along line profiles drawn through puncta showed a very high correlation between mCherry and Bassoon (Figure 4B; Pearson coeffcieint = 0.94; *P* < 0.0001). In contrast, there was no significant overlap between mCherry fluorescence and fluorescence obtained using an antibody against the post-synaptic marker PSD-95 (Figures 4C,D; Pearson coefficient = 0.17, *P* = 0.07). Images obtained using antibodies against vGlut1 showed very clear co-localization within a population of terminals (Figures 4E,F; Pearson coefficient = 0.96; *P* < 0.0001) suggesting that some, but not all, mCherry positive synapses were positive for vGluT1. A similar result was also obtained using an antibody against vGAT (Figures 4G,H; Pearson coefficient = 0.92; *P* < 0.0001). We conclude from these experiments that SyGCaMP2-mCherry is expressed in presynaptic boutons. We did not find any evidence from these immunohistochemical experiments or from multiphoton imaging of SyGCaMP2-mCherry for any expression in post-synaptic structures such as cell bodies, dendrites or dendritic spines. Expression was not exclusive to excitatory glutamatergic or inhibitory GABA-/glycinergic synaptic terminals since expression was observed in both types. Using multiphoton microscopy in living hippocampal slices, we were able to identify patterns of punctate expression at a wavelength of 920 nm that were virtually identical to those obtained using immunohistochemistry providing further evidence that SyGCaMP2-mCherry is expressed in presynaptic terminals.

**Figure 5:**
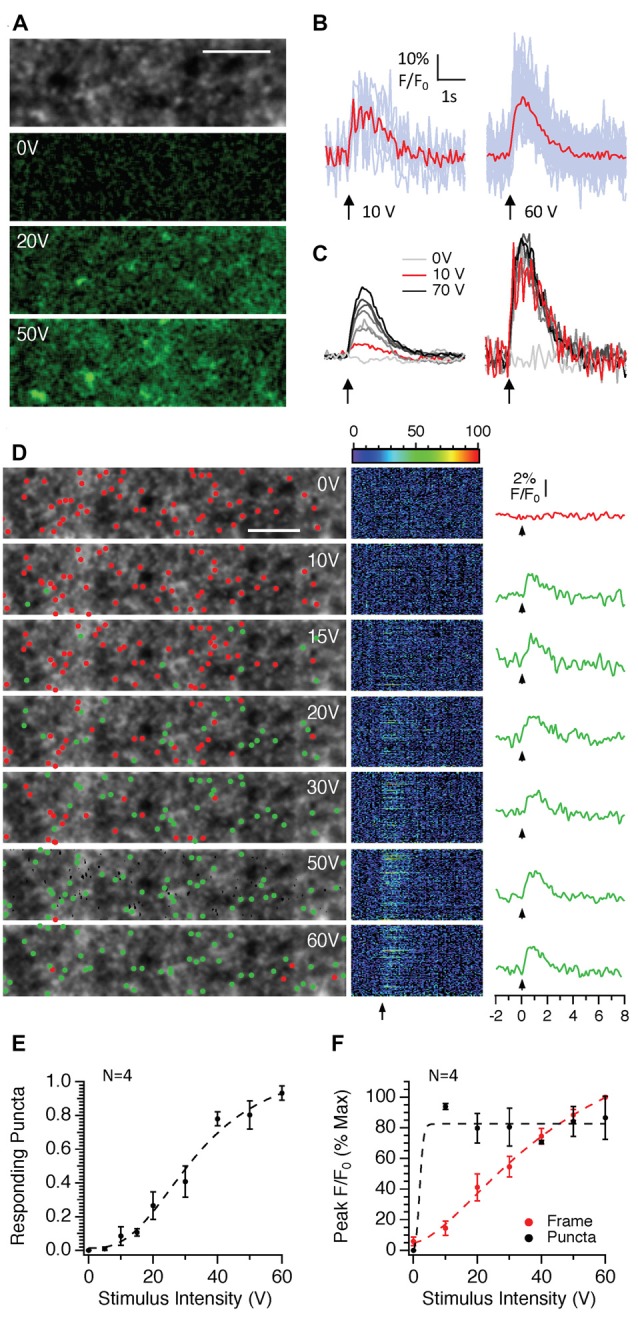
The effects of stimulus intensity on SyGCaMP2 responses in single presynaptic boutons in the CA1 region of the hippocampus. Images consisting of 512 pixels by 64 lines were scanned at rates of 98 ms and trains of 10 stimuli were delivered at 10 Hz at intensities ranging between zero and 70 volts. **(A)** The grayscale image illustrates the typical punctate expression observed in the *s. radiatum* of the CA1 hippocampal region. Shown below are images representing the difference in fluorescence during stimulation at intensities of 0, 20 and 50 V compared to baseline. A green look up table with identical brightness settings was applied to each image. The scale bar represents 10 μm. **(B)** Responding puncta were identified manually as described in the methods and changes in fluorescence over time measured and expressed as F/F_0_. Light blue traces represent responses from individual puncta and red traces show the average. Background subtracted responses from the entire image frame are shown on the left side of panel **(C)**. On the right are the average, background subtracted responses from responding puncta at each of the three intensities labeled. **(D)** Stacks of 100 images collected in response to stimulation at each intensity were background subtracted, averaged over time and a threshold segmentation approach (see “Materials and Methods” section for details) used to identify putative presynaptic boutons. Fluorescence responses from each of the identified regions are shown in the central panel with each pseudo-colored line representing a response over time from a single ROI. To the right, plotted with the same time scale, are averages of F/F_0_ measurements for those regions in which a response to stimulation was observed (green traces). Since there was no response at zero volts, the non-responding regions were averaged and displayed (red trace). Arrows indicate the onset of electrical stimulation. Green and red dots positioned at the point of maximum intensity for each ROI distinguish responding and non-responding ROIs respectively. The scale bar represents 10 μm. Panel **(E)** shows the number of punctate regions that respond to stimulation as a proportion of the total number of ROIs recorded from *n* = 4 slices from three different mice. Panel **(F)** compares the effect of stimulus intensity on the peak responses from responding puncta (black traces) compared to the entire background subtracted frame (red). The means and SEM are shown for *n* = 4 slices obtained from three separate mice. The dotted lines in panels **(E,F)** represent the lines of best fit using sigmoidal curve.

We next examined the effects of stimulus intensity on responses within single, manually identified puncta to examine whether the increases in fluorescence observed in Figure 2 were due to an increase in fluorescence within individual puncta and/or whether more puncta were recruited with increasing stimulus intensity. Figure 5A shows that as the stimulus intensity increased, the numbers of puncta responding increased. It was possible to measure clear responses from individual puncta at intensities above 10 V (Figure 5B). When fluorescence from the whole frame was measured, raising the stimulus intensity produced a near linear increase in fluorescence (Figures 5C,F). These results were entirely consistent with the widefield epifluorescence imaging results presented in Figure 2. Analysis of only those puncta that showed a discernible response at each stimulus intensity indicated that the average peak responses of responding puncta did not change significantly with stimulus intensity once the threshold for a detectable response was obtained. We confirmed this result using a different analysis method that used a Laplace Operator threshold segmentation method to automatically identify ROIs (Dorostkar et al., 2010). The number of responding puncta increased with stimulus intensity following a sigmoidal relationship (Figures 5D,E). Once the threshold for detection was reached the amplitude of responses from individual puncta did not change with intensity (Figures 5D–F). Increasing the stimulus intensity therefore recruits more boutons rather than increasing calcium entry per bouton. Given the all or none nature of action potentials, these results suggest that in the CA1 region, stimulus intensity does not have a particularly large effect on the failure rate of individual action potentials in response to stimulus trains because individual puncta do not change their response amplitudes with intensity.

**Figure 6:**
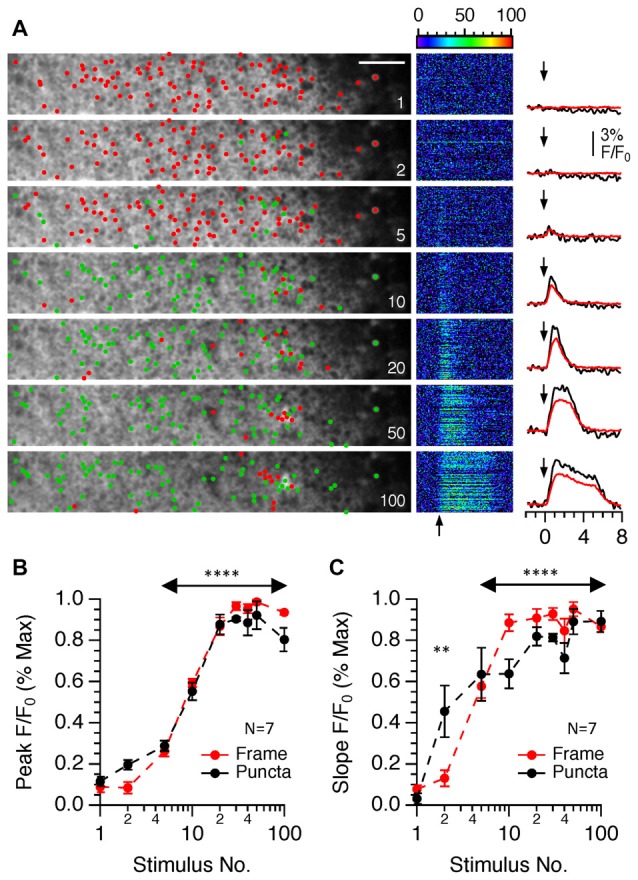
The effects of changing the stimulus number on SyGCaMP2 responses in single presynaptic boutons in the CA1 region of the hippocampus. Images consisting of 512 pixels by 64 lines were scanned at rates of 98 ms and trains of 1–100 stimuli were delivered at 20 Hz at a fixed intensity of 40 volts. **(A)** Data are illustrated in the same way as Figure 5D except that in the right-hand column the averages of responding puncta are shown to the right in black and the total frame fluorescence for each stimulus condition is shown in red. Arrows indicate the onset of electrical stimulation. Green and red dots positioned at the point of maximum intensity for each ROI distinguish responding and non-responding ROIs respectively. The scale bar represents 10 μm. Panel **(B)** shows the effect of stimulus number on the amplitude of the peak SyGCaMP2 responses for the total frame (red trace) and for only responding puncta (black trace). The effects on the initial slopes of responses are shown in panel **(C)**. A Kruskall-Wallace test was used to examine if there were any significant differences between peak and initial slopes of SyGCAMP2 responses to stimulus number. (**P = 0.0036; ****P < 0.0001). Data are presented as means and SEM for seven slices from four mice.

To test this, we next examined the effect of increasing the number of stimuli applied at a fixed intensity and frequency (Figure 6). The peaks of responses increased in an approximately linear manner up to 20 stimuli after which a plateau was reached (Figures 6A,B). Measurements of the initial slopes of responses revealed that it was possible to detect an increase in slope above baseline noise to only two stimuli although only very few responding puncta in a single field of view were detected (Figures 6A,C). In contrast to the effects of stimulus intensity, there was little difference between the responses of the entire frame and those of only responding puncta indicating that increasing the stimulus number does not recruit new synaptic boutons *per se* but it does increase the amount of calcium mobilized per bouton with each successive stimulus up to 20 stimuli, after which the response becomes saturated.

### Optical Measurement of Hippocampal CA1 Long-Term Potentiation

**Figure 7:**
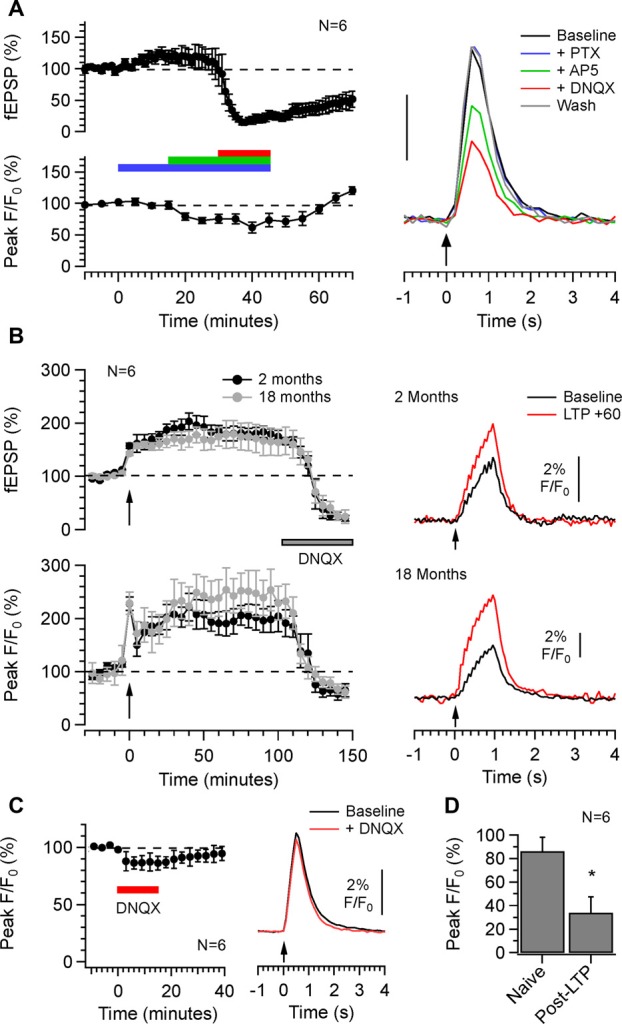
The effects of inhibition of inhibitory and excitatory synaptic transmission on SyGCaMP2 responses to SC-AC responses in CA1 and the measurement of long-term potentiation (LTP) following theta burst stimulation. **(A)** Pairs of stimuli were applied to SC-AC fibers every 10 s to record fEPSPs from the *s. radiatum*. At 5-min intervals, 10 stimuli were applied at 10 Hz and SyGCaMP2 fluorescence peak responses were measured. After 15 min, Picrotoxin (blue bars and traces), AP5 (green) and DNQX (red) were cumulatively applied, each for 15 min. After 45 min, all were washed off. fEPSP and SyGCaMP2 responses were expressed as a percentage of baseline F/F_0_ responses and plotted against time. Data represent the means and SEMs from six separate slices from three different mice. Traces to the right show SyGCaMP2 responses that were sampled before, during and after drug applications at the times indicated by colors. **(B)** fEPSPs were recorded every 10 s and the peak amplitudes of averaged responses plotted against time each minute. At 5-min intervals, bursts of 10 stimuli were applied at a frequency of 10 Hz and SyGCaMP2 peak responses from the *s. radiatum* measured. After 20 min, theta burst stimulation was applied and measurements of fEPSPs and SyGCaMP2 responses continued as before. After approximately 2 h, DNQX was applied to examine the proportion of the potentiated response that was presynaptic in origin. Experiments were performed in six young (<2 months of age) and six old (>18 months of age) animals. To the right are illustrated examples of traces taken before and 60 min after theta burst stimulation. **(C)** The effects of DNQX were tested on SyGCaMP2 peak responses in naive slices that had not previously undergone theta burst stimulation. The means and SEM of six separate experiments are shown. Representative traces recorded before and during DNQX application are shown on the right. **(D)** The effects of DNQX on SyGCaMP2 responses in naïve slices were compared with those from slices that had undergone LTP. The effect of DNQX was expressed as a percentage of the potentiated response rather than the original baseline. The means and SEM of six separate experiments from animals that were <2 months old in each case are shown. A Mann-Whitney U-test was used to test for statistically significant differences between potentiated and naive responses (*P = 0.022).

Our immunocytochemical analysis showed that SyGCaMP2-mCherry was present in both excitatory and inhibitory terminals. We recorded fEPSPs and SyGCaMP2 responses concurrently in CA1 in response to SC-AC activation and then added, sequentially, picrotoxin to block GABA/glycinergic transmission, AP5 to block NMDA receptors and DNQX to block AMPA receptors. Applications of PTX produced a small but statistically significant increase in the amplitudes of the N2 component of fEPSPs compared to baseline levels (*P* = 0.0442; *n* = 6; Friedman Test). SyGCaMP2 fluorescence responses were not significantly changed (*P* > 0.9999; *n* = 6; Figure 7A). This suggests that little if any of the total fluorescence signal originated from terminals that were downstream of an inhibitory, GABA-ergic or glycinergic synapse. Moreover, if picrotoxin resulted in an increase in reverberatory activity between CA1 and CA3, this was not detected by a change in SyGaMP2 fluorescence. In the presence of picrotoxin, inhibition of NMDA receptors with AP5 reduced N2 fEPSPs slightly and reduced SyGCaMP2 responses by around 15% of baseline although neither of these effects were statistically significant (*P* > 0.9999 and 0.4461 respectively). Subsequent inhibition of AMPA receptors produced a complete and statistically significant blockade of N2 fEPSPs as expected (*P* = 0.0013) but produced only a further 10% reduction of SyGCaMP2 responses (Figure 7A) that was not significantly different from baseline (*P* > 0.9999). Following washout of all of these inhibitors of synaptic transmission, SyGCaMP2 responses recovered and exceeded pre-drug baseline levels, suggesting that suppression of synaptic activity may lead to an up-regulation of presynaptic calcium influx or excitability.

Since part of the total SyGCaMP2 signal originates from presynaptic terminals that are post-synaptic to SC fibers, we reasoned that it may be possible to use this mouse model to record changes in synaptic strength through bouton recruitment within the CA1 region. Figure 7B shows the effects of theta burst stimulation on fEPSPs and SyGCaMP2 responses in hippocampal slices taken from young adult (2-month-old) and aged (18-month-old) mice. fEPSPs and SyGCaMP2 responses showed an increase in response size after theta burst stimulation that was maintained for more than 100 min. After this time, DNQX was added. fEPSPs were completely abolished as expected but SyGCaMP2 responses reduced to 66.6 ± 13.9% of the original baseline response after 15 min of application. In brain slices that had not previously undergone LTP, the effects of DNQX on SyGCaMP2 were tested (Figures 7C,D). In LTP naïve slices, SyGCaMP2 responses were reduced significantly to 86 ± 11.8% of baseline (*P* = 0.022; Mann-Whitney U test). This suggests that in the absence of LTP, only 14% of the total response comes from boutons that were synaptically connected via AMPA receptors. Once LTP had been induced, DNQX had a much larger effect reducing SyGCaMP2 responses to 33.9 ± 13.4% of the potentiated response (*P* = 0.022; Mann-Whitney U test). Whilst we cannot rule out some presynaptic contribution to this form of potentiation measured with SyGCaMP2, these data indicate that LTP is accompanied by a very substantial recruitment of boutons that are synaptically connected via AMPA receptors. Thus, although SyGCaMP2 is expressed in presynaptic terminals, it is possible to use the SyG37 mouse to monitor patterns of synaptic activation and changes in synaptic connectivity.

### Characteristics of CA3 Responses to Dentate Gyrus Stimulation

**Figure 8:**
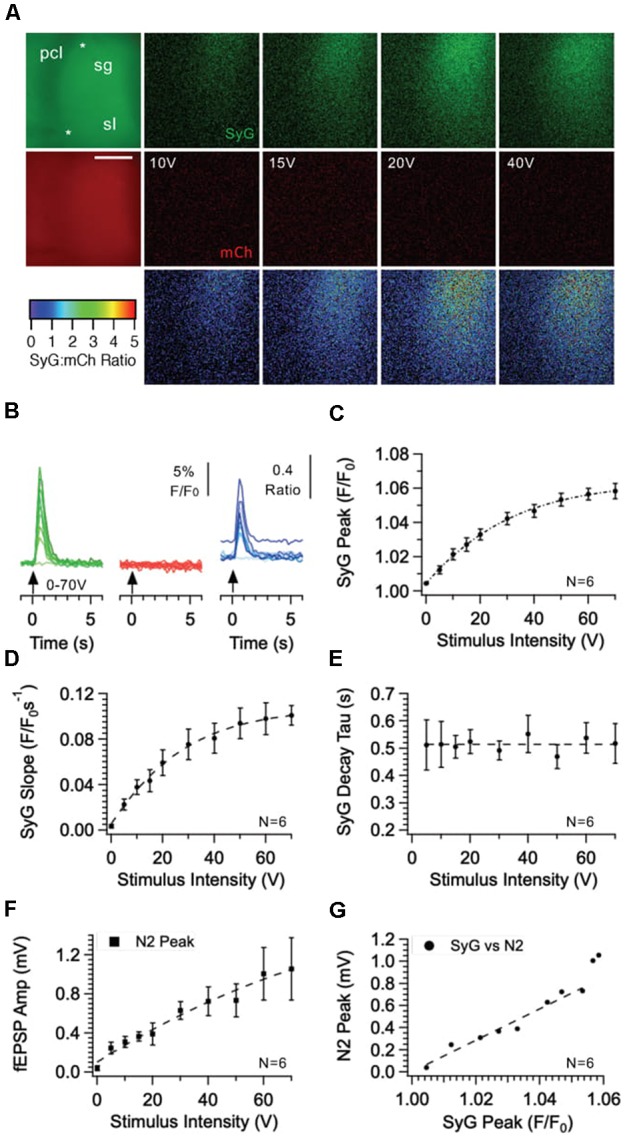
Effect of stimulus intensity on fluorescence responses recorded from the CA3 region of the hippocampus in SyGCaMP2-mCherry expressing mice. Bursts of 10 stimuli over a range of intensities were delivered at 20 Hz via a patch pipette placed *s. granulosum* of the dentate *gyrus*. Images of SyGCaMP2 (green) and mCherry fluorescence (red) are shown along with the ratio of the two and images illustrating the difference in fluorescence for each fluorophore and their ratio before and during stimulation at each intensity labeled **(A)**. The positions of the stimulating and recording electrodes are marked with upper and lower asterisks respectively. Abbreviations: pcl; pyramidal cell layer; sg; *s. granulosum; sl; stratum lucidum*. The horizontal scale bar represents 100 μm. Responses over time are shown **(B)** for SyGCaMP2 and mCherry fluorescence extracted from ROIs placed over the *s lucidum (sl)*. The ratios of SyGCaMP2:mCherry fluorescence at each intensity are shown in blue. Increasing intensities are depicted with darker hues. The mean and SEM peak **(C)**, initial slope **(D)** and decay time constant **(E)** of SyGCaMP2 fluorescence responses and the N2 component of fEPSPs **(F)** are plotted against intensity. The relationship between SyGCaMP2 and N2 peak responses is shown in panel **(G)**. A line of best fit was plotted for values measured below 50 V where the relationship was linear. Data were obtained from six separate hippocampal slices taken from four different mice.

**Figure 9:**
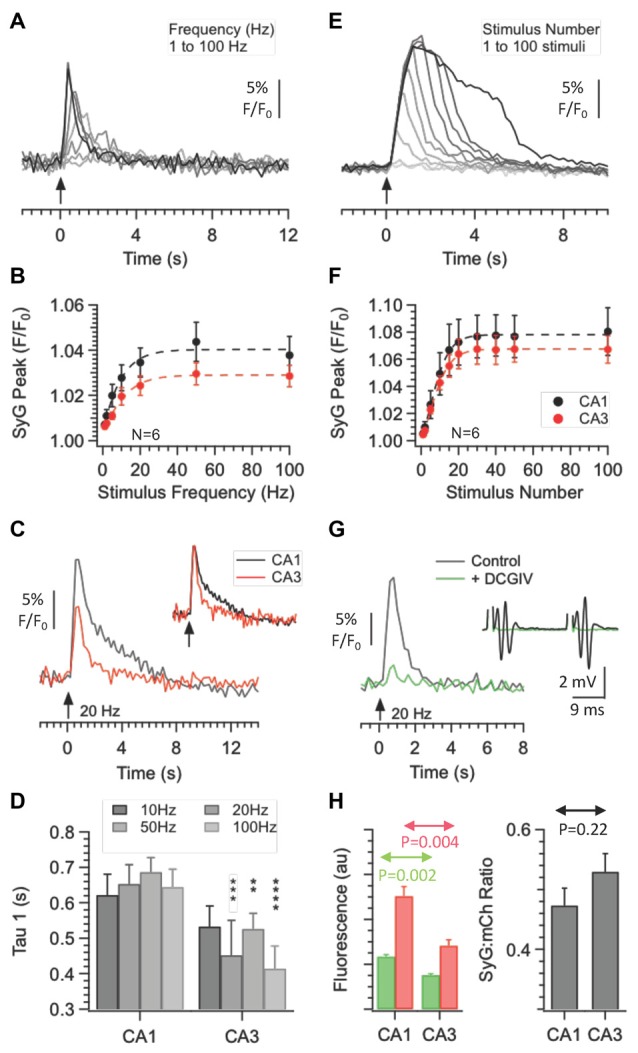
Effect of stimulus frequency and number on fluorescence responses recorded from the CA3 *s. lucidum* and CA1 *s. radiatum* of SyG37 mice. Trains of 20 stimuli were applied at a fixed voltage at frequencies between 1 Hz and 100 Hz. In panel **(A)** responses over time are shown for SyGCaMP2 fluorescence extracted from ROIs placed over the *s. lucidum*. **(B)** The means and SEM for the peak responses are shown alongside data recorded from CA1 (*n* = 6 slices from four different mice in each case). Panel **(C)** Examples of traces recorded at 20 Hz from CA3 and CA1 are shown. The inset shows the two responses scaled to the peak to highlight the different decay time courses. **(D)** The decay phases of responses were fitted with double exponential curves and the faster value of the time constant tau plotted against frequency at each region. The means and SEM from 18 separate experiments are shown. (***P = 0.0015 (20 Hz); **P = 0.0085 (50 Hz) and ****P = 0.0003 (100 Hz); Kruskall-Wallace Test with Dunn’s multiple comparisons; *n* = 18 in each case). Panel **(E)** provides an example of CA3 responses to increasing stimulus numbers delivered at a fixed intensity at 20 Hz. Data are plotted to the same scale as that in panel **(A)**. Data pooled from six separate slices from four different mice are shown in panel **(F)**. **(G)** Applications of the mGluR2 agonist DCGIV reduced both SyGCaMP2 responses and fEPSPs recorded in *s. lucidum*. The absolute fluorescence of both mCherry (red bars) and SyGCaMP2 (green bars) under baseline conditions are shown **(H)** along with the ratio of SyG:mCherry fluorescence. The means and SEM of six separate experiments from six different mice are shown (*P* = 0.002 (SyGCaMP2); *P* < 0.004 (mCherry); *P* = 0.22 (Ratio) Mann-Whitney U-test).

We next examined SyGCaMP2 responses within the CA3 region (see Figures 8, 9). Stimulating and recording electrodes were positioned to produce fEPSPs that displayed significant levels of paired pulse facilitation. At the end of experiments, we verified that responses were abolished upon application of the mGluR2 agonist DCGIV (see Figure 9G). These two characteristics are indicative of mossy fiber responses (Nicoll and Schmitz, 2005). SyGCaMP2 responses within the CA3 region displayed properties that were very similar to those observed in CA1. In response to increasing stimulus intensities, the patterns of responses in CA3 were qualitatively similar to those in CA1 but slightly, although not significantly smaller (*P* > 0.9999; Mann-Whitney U-test; compare Figures 2, 8). Responses to changes in frequency and stimulus number were also qualitatively similar to those in CA1 but consistently had smaller peak responses and faster decay times (Figures 9A–F). Only the decay times at frequencies above 10 Hz were significantly different (Figure 9D). SyGCaMP2 responses, along with the fEPSPs, were completely blocked by applications of DCGIV (Figure 9G).

To establish whether differences in expression levels and/or background fluorescence may have contributed to these small differences in peak responses between CA1 and CA3, we measured absolute fluorescence levels in CA1 and CA3 and autofluorescence of age-matched slices from WT animals to estimate background autofluorescence. Figure 9H shows that expression of SyGCaMP2 and mCherry was significantly higher in CA1 than in CA3. The ratio of SyGCaMP2:mCherry, which is proportional to absolute calcium, was not significantly different, suggesting that the concentration of residual calcium in CA1 and CA3 terminals is similar. Background fluorescence was not significantly different between CA1 and CA3 regions but it was proportionally lower in CA1 because fluorescence of SyGCaMP2 and mCherry were higher. Using these values for background subtraction we found that the absolute peak values of CA1 and CA3 SyGCaMP2 fluorescence in response to stimulation were both increased and although CA1 responses remained higher, the size of the difference decreased and was still not statistically significantly different from those in CA3.

**Figure 10:**
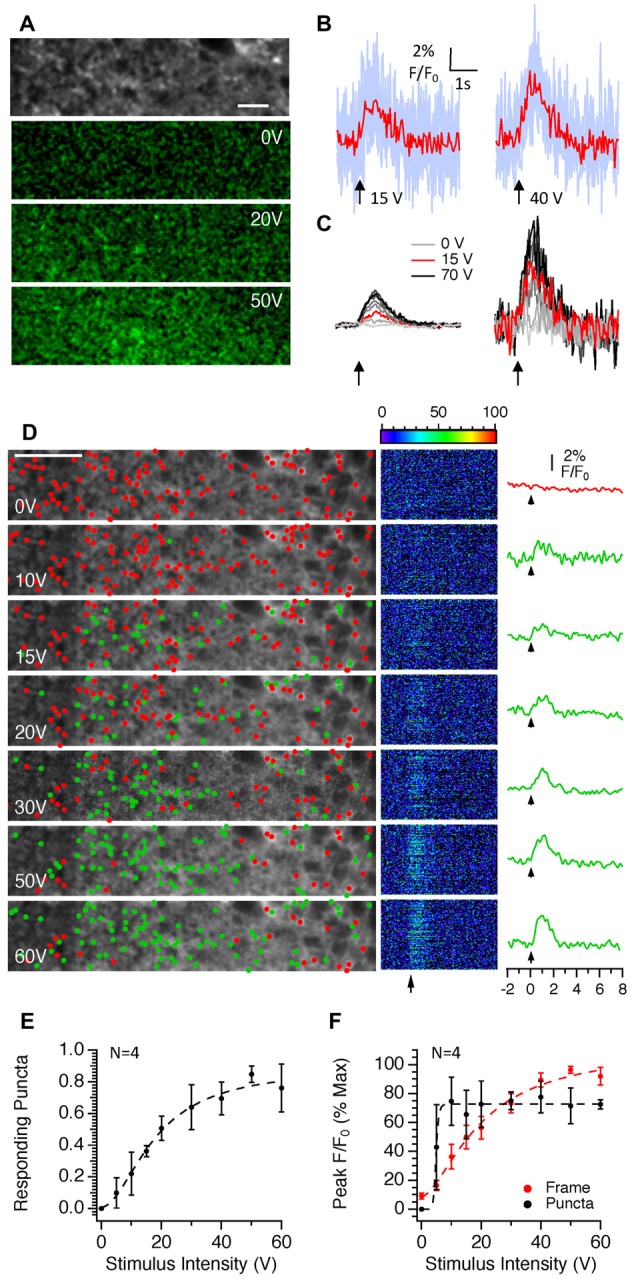
Effect of stimulus intensity on SyGCaMP2 responses in single presynaptic boutons within CA3. Images consisting of 512 pixels by 64 lines were scanned at rates of 98 ms and trains of 10 stimuli were delivered at 10 Hz at intensities ranging between zero and 70 volts. Panels **(A–F)** are presented in the same way as those in Figure 5.

**Figure 11:**
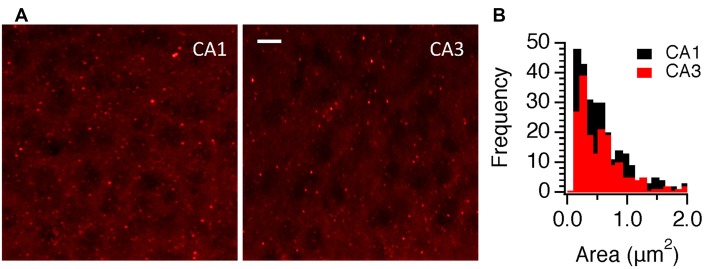
Expression density of SyGCaMP2-mCherry in CA1 and CA3 regions of the hippocampus. Panel **(A)** compares the punctate patterns of staining in hippocampal sections obtained from SyG37 mice using an antibody raised against mCherry in CA1 and CA3 regions. The scale bar represents 10 μm. **(B)** The frequency distributions of identified puncta according to their areas are shown.

Using multiphoton imaging where it was easy to background subtract, we examined CA3 responses in individual boutons (Figure 10) and compared the results with responses from CA1 (Figure 5). As with widefield epifluorescence measurements, we did not find differences in the peak responses from single boutons but SyCaMP2 responses decayed faster in CA3. From these experiments, we noticed that the number of boutons responding to stimulation appeared reduced in CA3 compared to equivalent responses in CA1. We therefore returned to immunocytochemical data and compared the density of boutons in each region. As shown in Figures 11A,B, the density of mCherry labeled boutons was higher in CA1 by a factor of 1.5. This suggests either that SyGCaMP2-mCherry is expressed in more CA1 boutons than CA3 boutons or that the total density of presynaptic boutons per unit volume is higher in CA1. Therefore, the most likely explanation for the larger responses in CA1 than CA3 in widefield fluorescence experiments is that more boutons contribute to the total response. Terminals in CA3 were however able to recover fluorescence levels to baseline more quickly after stimulation than those in CA1 and may indicate that CA3 terminals are better able to homeostatically control calcium levels after activation than those in CA1.

## Discussion

### Expression and Subcellular Location of SyGCaMP2-mCherry

We generated a transgenic mouse line that expressed the ratiometric GECI SyGCaMP2-mCherry under the control of the Thy1–2 promotor and targeted to presynaptic terminals by fusing the GECI to synaptophysin. Expression of mCherry was co-localized with GCaMP2 and both fluorophores were present throughout the brain but with particularly high levels of expression in the hippocampus, cortex, thalamus as well as the cerebellum (not shown). At high magnifications, expression was clearly punctate and we found no evidence for expression in post-synaptic structures such as soma or dendrites. Immunocytochemical analysis of expression within the hippocampus using antibodies raised against mCherry, bassoon, VGlut1, VGAT and PSD-95 demonstrated that there was no statistically significant co-localization of mCherry with the post-synaptic marker PSD95. It was statistically significantly co-localized with the presynaptic marker bassoon and with both VGlut1 and VGAT indicating that SyGCaMP2-mCherry is found in both excitatory and inhibitory presynaptic terminals.

### Sensitivity to Electrical Activation

GECIs can be used to detect neuronal activity provided their responses to calcium signals have sufficient dynamic range, sensitivity and kinetic speed. Although calcium sensors respond too slowly to resolve the kinetics of action potentials, it is possible to estimate ongoing neuronal firing rates as the calcium signals associated with neuronal activity summate. Within the CA1 region, electrical stimulation of the SC-AC pathway produced robust increases in GCaMP2 fluorescence in CA1 with no change in the fluorescence of mCherry. With widefield epifluorescence measurements, it was possible to detect responses to a single stimulus. GCaMP2 responses increased with the intensity of stimulation and there was a significant correlation between the amplitudes of concurrently recorded field potentials. Fluorescence responses measured at a fixed stimulus intensity and frequency increased linearly with stimulus number up to 10 stimuli, after which responses started to plateau. These results are entirely consistent with those obtained for SyGCaMP2 in cultured hippocampal neurones (Dreosti et al., 2009). With fixed numbers of stimuli, responses increased with stimulus frequency up to 10 Hz. At frequencies above this, responses did not increase further in amplitude. Measurement of the initial rising phase of SyGCaMP2 responses showed linear increases up to 20 Hz and 20 stimuli. The plateau observed from measurements of the peak response was not simply due to saturation of the sensor because applications of increased concentrations of extracellular calcium in the presence of the calcium ionophore ionomycin produced much larger peak responses than those elicited synaptically (data not shown). In the CA3 region, SyGCaMP2 the patterns of responses originating from or driven by MF activation were qualitatively very similar to those in CA1 except that the peak amplitudes and initial slopes of responses were slightly smaller.

Measurements of fluorescence recovery after stimulation were different between CA1 and CA3. Whereas responses slowed as the intensity of stimulation increased in CA1, the time course for fluorescence decay in CA3 did not change with stimulus intensity and recovery to baseline was significantly quicker over the entire range of frequencies tested. The differences in the rates of SyGCaMP2 fluorescence recovery between CA1 and CA3 may well reveal differences in the abilities of CA1 and CA3 synapses to homeostatically maintain calcium levels after activation.

Widefield epifluorescence measurements represent an ensemble recording of many thousands of boutons from a potentially large volume of neuronal tissue. Without optical sectioning, it was difficult to separate the fluorescence from individual boutons from background fluorescence originating from out of focus boutons. Using multiphoton excitation at 920 nm, it was possible to visualize single boutons that responded to electrical stimulation. In the CA1 region, and in contrast to widefield epifluorescence measurements, the fluorescence of single responding boutons did not vary linearly with stimulus intensity. The numbers of boutons that responded did increase with stimulus intensity. Thus, at a given frequency and number of stimuli, increasing the intensity of stimulation recruits more boutons. A similar effect was seen in CA3. When the amplitudes of responses from single boutons in CA3 were compared with those in CA1, multiphoton recordings showed that there was no difference. Estimation of the density of boutons in CA3 compared to CA1 revealed that the numbers of mCherry expressing boutons in CA3 was lower. It is likely therefore that the smaller responses measured in CA3 using widefield imaging reflected a reduced level of expression or a lower density of boutons compared to CA1. Measurements from single boutons confirmed the difference in decay time constants providing further evidence for a fundamental difference in calcium homeostatic mechanisms between CA1 and CA3 presynaptic boutons.

Using multiphoton microscopy, it was not possible to reliably detect responses to a single electrical stimulus in hippocampal brain slices from SyG37 mice. We could reliably detect responses in single puncta to two or five stimuli (Figure 6). As discussed previously (Dreosti et al., 2009), targeting GCaMP2 to the active zone of synapses by fusing it to the intracellularly facing terminal of synaptophysin increases its sensitivity compared to GCaMP2 (Hendel et al., 2008) because the sensor is confined to a micro-environment where calcium is increased rapidly to very high levels. Dreosti et al. (2009) were able to observe responses to single stimuli in single boutons but these results were obtained in hippocampal cultures using camera-based detection methods. Using similar methods with a version of SyGCaMP2-mCherry expressed in primary hippocampal cultures we were also able to detect responses to single stimuli (data not shown) suggesting that the addition of mCherry did not alter the sensitivity of SyGCaMP2-mCherry compared to SyGCaMP2. Our inability to detect responses to single stimuli in single boutons in brain slices most likely reflected a difference in expression levels compared to transiently transfected primary cultures. With multiphoton microscopy, it was possible to measure responses from the same boutons in slices several times reliably before samples bleached. By minimizing the laser power, we were able to scan single boutons 10–20 times for several seconds. Higher laser powers allowed faster rates of imaging and improved sensitivity but at the expense of the duration of the recording. We could image at much faster speeds using widefield epifluorescence from ensembles of boutons. Under these conditions it was possible to detect responses to single stimuli (see Figures 3A,D). The biggest hurdle we encountered for making multiple measurements from the same, identified boutons over prolonged periods of time was movement of the tissue. However, the density of expression in CA1 and CA3 is sufficiently high to allow multiple measurements to be made over time but not necessarily from precisely the same populations of boutons.

One disadvantage of using a Thy1–2 promotor is that there is no control over which populations of neurones express the GECI. In response to SC-AC stimulation in CA1, responses originated from both the SC-AC terminals themselves but also presynaptic terminals of other neurones connected mono or poly-synaptically. Inhibition of AMPA receptors completely blocked the N2 component of CA1 fEPSP responses and reduced SyGCaMP2 peak responses by about 14%. This indicates that ~86% of the total SyGCaMP2 fluorescence originates directly from SC-AC terminals. Inhibition of GABA/Glycinergic synapses increased the amplitude of SyGCaMP2 responses and N2 fEPSPs although the effect was small and not statistically significant. Intrinsic inhibitory interneurons within CA1 provide a tonic inhibition of Pyramidal cell activity. It is likely therefore that the small increase in fluorescence we observed was due to a lifting of this tonic inhibition and a recruitment of additional excitatory connections and their presynaptic boutons. Inhibition of NMDA receptors produced little effect on either SyGCaMP2 responses or N2 fEPSPs. This is also consistent with the fact that NMDA receptors contribute only little to synaptic transmission at CA1 under normal conditions of synaptic activation. It was interesting to note that on washout of this cocktail of synaptic inhibitors, SyGCaMP2 responses recovered and exceeded baseline levels. The reasons for this are not yet apparent but it is interesting to note that in hippocampal cultures, chronic suppression of synaptic activity using TTX leads to a homeostatic increase in presynaptic calcium influx and transmitter release (Zhao et al., 2011).

Signals recorded from SyG37 mice can be used to visualize the spatial patterns of fiber activation when synaptic transmission is blocked. This is similar to the N1 response or the fiber volley but with the added advantage of producing a spatial map of which fibers and terminals are activated. When synaptic transmission is intact, the SyGCaMP2 signals provide a spatial map of the entire active network. It was possible to modulate the strength of synaptic transmission within CA1 by inducing LTP through theta burst activation of SC-AC fibers. LTP of fEPSPs was accompanied by an increase in the size of SyGCaMP2 responses. The proportional effect of DNQX increased significantly after the induction of LTP compared to LTP naïve slices. Although we cannot rule out some presynaptic contribution to LTP, these results clearly demonstrate that a very significant proportion of the potentiated response was due to the recruitment of additional presynaptic terminals that were connected via AMPA receptors.

A limitation of using GECIs to detect neuronal activity is their slow time course of fluorescence changes compared to the actual underlying calcium transients. Signals measured using SyGCaMP2 (Dreosti et al., 2009; Zhao et al., 2011) or SyGCaMP2-mCherry are highly temporarily filtered but even so, they can still be used to detect individual action potentials. The addition of mCherry allows the expression levels of the sensor to be estimated and so calibrate residual calcium as an index of ongoing activity of ensembles of boutons. Using this method, we found no significant differences between residual calcium in CA1 and CA3 boutons. Expression levels in SyG37 mice did not change significantly with age nor did we find any evidence for altered behavior or longevity. This model can therefore be used compare synaptic properties during ageing and crossed with other transgenic models to examine, for example, the effects of neurodegenerative conditions on dynamic and absolute presynaptic calcium signaling in brain slices or in freely moving mice or in head fixed *in vivo* models. With low magnifications, it is possible to measure ensemble synaptic responses over large spatial areas with very high signal to noise ratios and when combined with electrophysiological recordings, potential pre- and/or post-synaptic drug targets identified. This model may prove particularly useful for investigating the effects of receptors and second messenger pathways that are thought to influence presynaptic calcium signaling and excitability.

In the last few years significant improvements have been made to the properties of GECIs, including GCaMP6 which is much brighter than GCaMP2 and which produce larger changes in calcium dependent fluorescence (Chen et al., 2013). Given that the approach of targeting GECIs to presynaptic terminals increases the sensitivity of GCaMP2 to detect neuronal activity (Dreosti et al., 2009), we predict that this same approach applied to GCaMP6 or future and even further improved GECIs would easily allow detection of single action potentials in single boutons. Combining this or a related sensor with a spectrally distinct indicator of post-synaptic calcium, transmitter release or optogenetic modulators of membrane potential would provide a further useful tool for assessing synaptic transmission using optical approaches.

## Ethics Statement

This work was carried in in accordance with the UK Animals (Scientific Procedures) Act 1986 and with the approval of the University of Leicester Animal Welfare and Ethical Review Body.

## Author Contributions

IA-O and DP carried out experimental work, analyzed data and helped to write the manuscript. MM generated the transgenic mouse and helped to write the manuscript. MP-G, AO and RT contributed to the experimental work. NH conceived the project, contributed to experimental work and data analysis, wrote software for analysis and drafted the manuscript.

## Conflict of Interest Statement

The authors declare that the research was conducted in the absence of any commercial or financial relationships that could be construed as a potential conflict of interest.
