# Corrigendum: Imaging Calcium in Hippocampal Presynaptic Terminals With a Ratiometric Calcium Sensor in a Novel Transgenic Mouse

**DOI:** 10.3389/fncel.2019.00194

**Published:** 2019-05-15

**Authors:** Ibrahim Al-Osta, Mariusz Mucha, Daniel Pereda, Marta Piqué-Gili, Albert E. Okorocha, Roisin Thomas, Nicholas A. Hartell

**Affiliations:** Department of Neuroscience, Psychology and Behaviour, University of Leicester, Leicester, United Kingdom

**Keywords:** presynaptic terminals, calcium imaging, transgenic mice, hippocampus, CA1, CA3, long-term potentiation

In the original article, there was a mistake in Figure 8 as published. During the revision process, panels B-G from Figure 2 were incorrectly duplicated into panels B-G of Figure 8. The data for these figures were correct in the original submission. The corrected Figure 8 appears below. The authors apologize for this error and state that this does not change the scientific conclusions of the article in any way. The original article has been updated.

**Figure 8:**
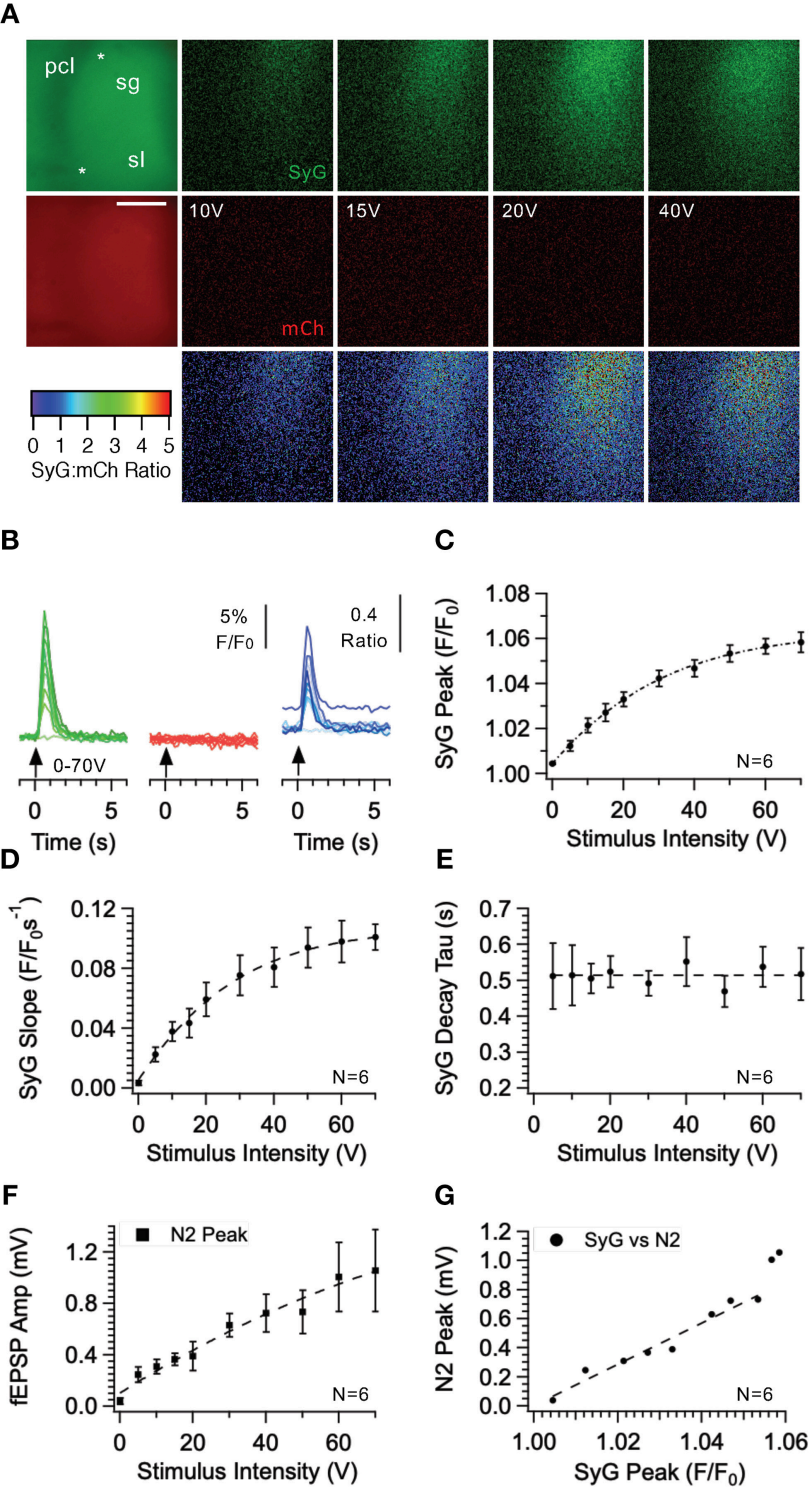
Effect of stimulus intensity on fluorescence responses recorded from the CA3 region of the hippocampus in SyGCaMP2-mCherry expressing mice. Bursts of 10 stimuli over a range of intensities were delivered at 20 Hz via a patch pipette placed *s. granulosum* of the dentate *gyrus*. Images of SyGCaMP2 (green) and mCherry fluorescence (red) are shown along with the ratio of the two and images illustrating the difference in fluorescence for each fluorophore and their ratio before and during stimulation at each intensity labeled **(A)**. The positions of the stimulating and recording electrodes are marked with upper and lower asterisks respectively. Abbreviations: pcl; pyramidal cell layer; sg; *s. granulosum; sl; stratum lucidum*. The horizontal scale bar represents 100 μm. Responses over time are shown **(B)** for SyGCaMP2 and mCherry fluorescence extracted from ROIs placed over the *s lucidum (sl)*. The ratios of SyGCaMP2:mCherry fluorescence at each intensity are shown in blue. Increasing intensities are depicted with darker hues. The mean and SEM peak **(C)**, initial slope **(D)** and decay time constant **(E)** of SyGCaMP2 fluorescence responses and the N2 component of fEPSPs **(F)** are plotted against intensity. The relationship between SyGCaMP2 and N2 peak responses is shown in panel **(G)**. A line of best fit was plotted for values measured below 50 V where the relationship was linear. Data were obtained from six separate hippocampal slices taken from four different mice.

